# Endoscopic ultrasonography‐guided removal of a stent that had migrated into the pancreas post‐pancreaticojejunostomy: A case report

**DOI:** 10.1002/deo2.70096

**Published:** 2025-03-11

**Authors:** Satoshi Kajitani, Kazuyuki Matsumoto, Kentaro Oki, Akihiro Matsumi, Kazuya Miyamoto, Yuki Fujii, Daisuke Uchida, Koichiro Tsutsumi, Shigeru Horiguchi, Motoyuki Otsuka

**Affiliations:** ^1^ Department of Gastroenterology and Hepatology Okayama University Hospital Okayama Japan

**Keywords:** endoscopic introducer, endoscopic ultrasonography‐guided pancreatic duct drainage, endosonographically/EUS‐guided created route, EUS‐guided interventions, internal stent

## Abstract

A 64‐year‐old woman had undergone subtotal stomach‐preserving pancreaticoduodenectomy for locally advanced pancreatic head cancer. She had an uneventful postoperative course with no recurrence. However, approximately 18 months after surgery, she presented with recurrent abdominal pain. Although contrast‐enhanced computed tomography abdominal radiographs showed internal stent migration to the residual pancreas, dilatation of the tail side of the pancreatic duct was observed. The impaired internal stent was considered to be the cause of the abdominal pain. An attempt to remove the stent via balloon‐assisted endoscopy was unsuccessful as the pancreaticojejunostomy site could not be reached. Consequently, endoscopic ultrasonography‐guided pancreatic duct drainage was performed, and a plastic stent was placed through the jejunal site to the stomach. Two months later, the endosonographically/endoscopic ultrasonography‐guided created route was dilated, and an endoscopic introducer was inserted into the pancreatic duct. Biopsy forceps were advanced through the sheath, allowing the successful removal of the stent by direct grasping. The symptoms of the patient improved, and she was discharged without complications.

## INTRODUCTION

Internal stents are often placed at the pancreatojejunostomy site following pancreaticoduodenectomy (PD) to prevent postoperative complications such as pancreatic fistulas.^1^ While these stents are usually expelled spontaneously,^2^ retained stents can lead to complications in rare cases, including pancreatitis. Balloon‐assisted endoscopy is typically used to remove retained stents. However, postoperative adhesions and anatomical alterations disrupt access to the pancreatojejunostomy site.

Endoscopic ultrasonography‐guided pancreatic duct drainage (EUS‐PD) has been reported as an effective approach for pancreatic duct assessment in patients with surgically reconstructed intestinal anatomy.^3^ EUS‐PD is particularly useful in cases where conventional balloon‐assisted endoscopy fails. Notably, several treatments using a trans endosonographically/EUS‐guided created route (ESCR) have been reported.

Recently, a newly developed endoscopic introducer with a dual‐layer structure, consisting of an inner sheath and an outer sheath, enabled device insertion up to 5.7Fr diameter after the removal of the inner sheath. This device is useful for biopsies of bile duct lesions and for overcoming challenging cases.^4^, ^5^, ^6^, ^7^, ^8^ Here, we report a case of successful removal of an internal stent that had migrated to the pancreatic duct following PD by employing an ESCR that involved using a newly developed endoscopic introducer.

## CASE REPORT

A 64‐year‐old woman was diagnosed with an unresectable locally advanced pancreatic head cancer. She underwent chemotherapy with modified folinic acid, fluorouracil, irinotecan, and oxaliplatin (mFOLFIRINOX) for 18 months, which achieved local tumor control without the emergence of distant metastases. Subsequently, radiotherapy was administered, followed by a subtotal stomach‐preserving PD. Approximately 18 months later, the patient presented with recurrent, persistent abdominal pain that lasted for approximately 1 month, prompting her to seek medical attention. Laboratory test results showed no evidence of an inflammatory response or elevated pancreatic enzyme levels (white blood cell 1.69 × 10^3^/µL, C‐reactive protein 0.04 mg/dL, amylase 94 mg/dL, and lipase 13 U/L). However, the glycosylated hemoglobin A1c levels of the patient worsened compared to 2 months prior measurement (8.2% vs. 7.4%, respectively).

**FIGURE 1:**
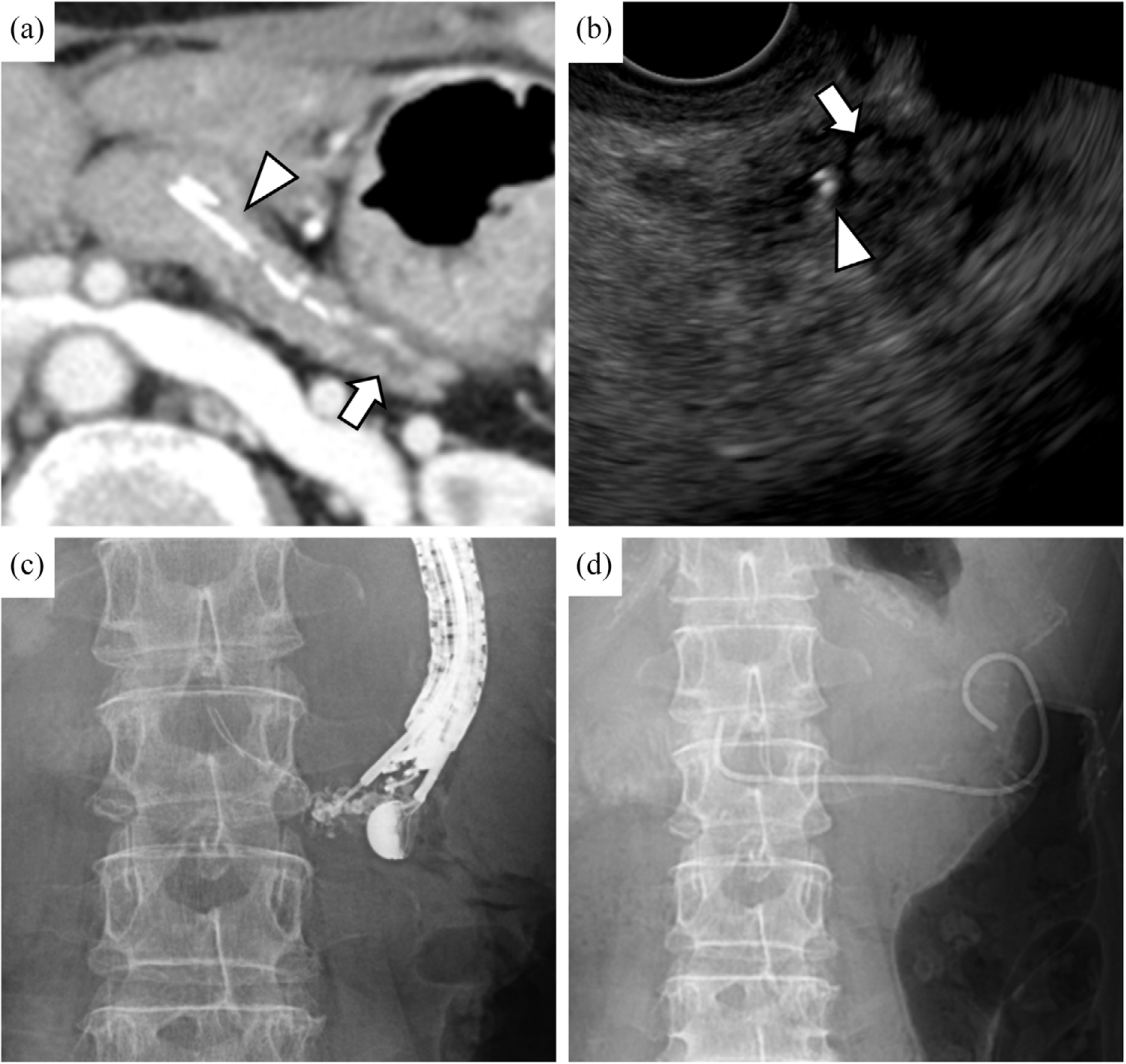
Radiographs showing a stent that had migrated into the pancreatic duct. (a) Using contrast‐enhanced computed tomography, an internal stent (arrowhead) was observed within the main pancreatic duct, along with dilation of the tail side of the main pancreatic duct (arrow). (b) Employing endoscopic ultrasonography, an internal stent (arrowhead) and debris echoes (arrow) were identified within the pancreatic duct. (c) The pancreatic duct was punctured transgastrically using a 19G needle for fine‐needle aspiration. (d) A 6Fr double pigtail plastic stent was placed with one pigtail positioned in the jejunum and the other in the stomach.

Abdominal contrast‐enhanced computed tomography revealed a retained internal stent in the pancreatic duct with dilation of its tail side (Figure 1a). EUS identified the stent and debris echoes, suggesting an obstruction of pancreatic juice outflow (Figure 1b). The abdominal pain was attributed to obstruction of pancreatic juice outflow caused by the internal stent. First, double‐balloon enteroscopy (DBE) was conducted using a short‐type DBE (EI‐580BT: working length 1550 mm; Fujifilm) which failed to access the pancreatojejunostomy site due to postoperative intestinal adhesions. A long‐type DBE (EN‐580T: working length 2000 mm; Fujifilm) was then attempted but remained unsuccessful even for 110 min. Consequently, stent removal via an EUS‐PD route was planned.

The pancreatic duct was punctured transgastrically using a 19G needle (EZ Shot 3 Plus; Olympus), followed by the insertion of a 0.025‐inch guidewire (VisiGlide 2; Olympus) that was advanced across the pancreatojejunostomy site (Figure 1c). After dilating the puncture site using a drill‐type endoscopic dilator (Tornus ES; Asahi Intecc), a 6Fr double‐pigtail plastic stent (Zimmon Biliary Stent; Cook Medical) was placed, considering the 2 mm pancreatic duct and the risk of deviation. One pigtail was positioned in the jejunum and the other in the stomach (Figure 1d).

**FIGURE 2:**
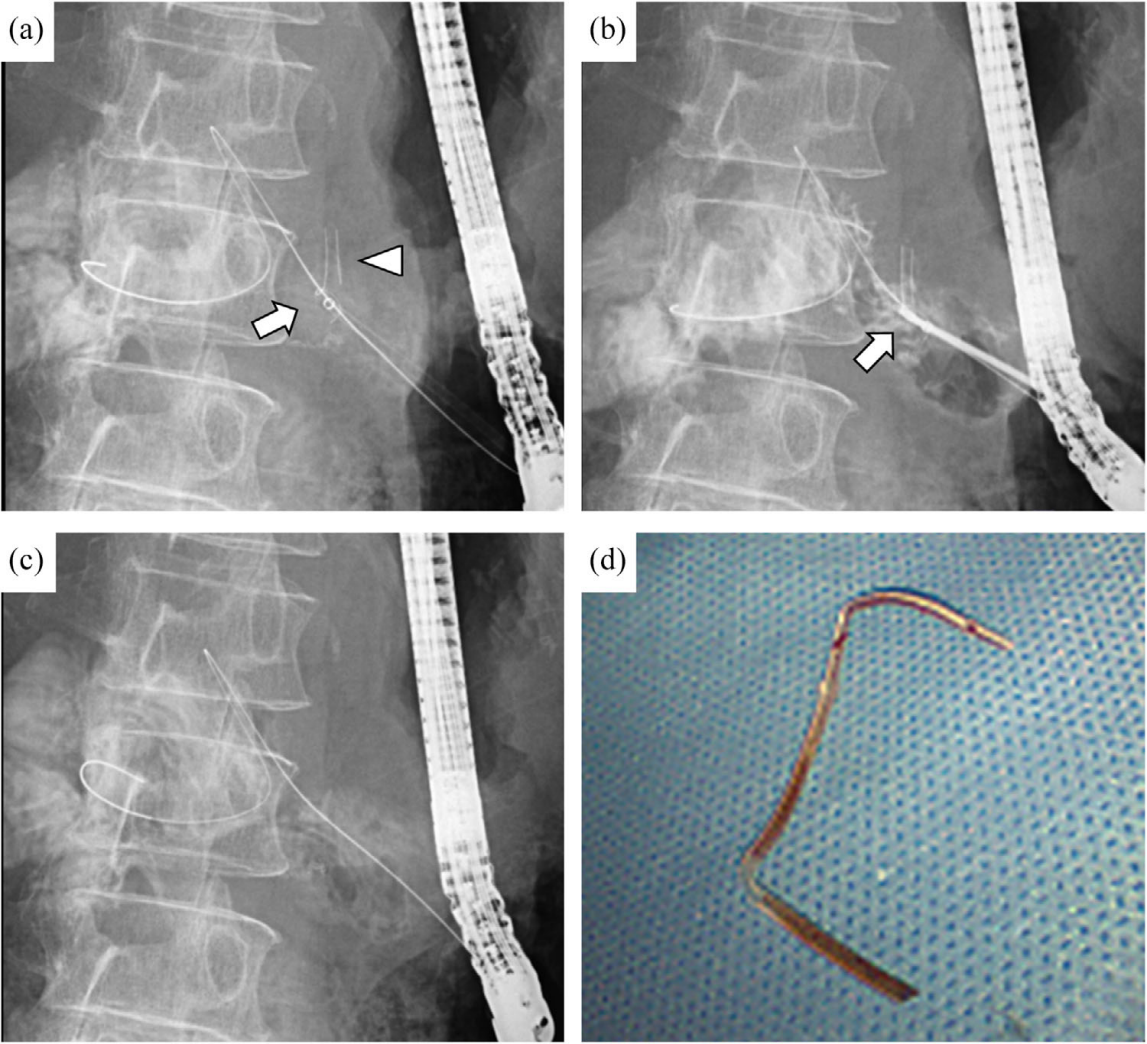
Radiographs showing the removal of a stent that had migrated into the pancreatic duct. (a) An endoscopic introducer (arrow) was placed near the internal stent (arrowhead) within the pancreatic duct. (b) Biopsy forceps (arrow) were inserted into the endoscopic introducer to directly grasp the internal stent. Both the introducer and the stent were retracted together. (c) The internal stent was successfully removed without any remnants. (d) Photograph of the removed internal stent.

Approximately 2 months later, the double‐pigtail plastic stent was removed using forceps after securing the route with a guidewire. The ESCR was dilated to 4 mm using a balloon dilator (REN; KANEKA Medix), and an endoscopic introducer (EndoSheather; Piolax) was placed within the pancreatic duct (Figure 2a). Bbiopsy forceps (1.8 mm outer cup diameter, Radial Jaw 4; Boston Scientific) were then inserted to directly grasp and remove the internal stent (Figure 2b–d). On the same day, to prevent post‐procedural pancreatitis, a 7Fr single‐pigtail plastic stent (Through & Pass TYPE IT; KANEKA Medix) was placed and the patient was discharged without any complications. The pancreatic duct stent was removed approximately one month later, leading to symptom improvement.

## DISCUSSION

In PD, internal stents are often placed in the pancreatic duct to prevent postoperative pancreatic fistulas and to maintain ductal patency in early anastomotic edema cases.^1^ According to Kadowaki et al., internal stents are spontaneously expelled in most cases, with a reported median time to expulsion of 454 days.^2^ Shin et al. reported that among 88 patients with internal stent placement after pancreaticobiliary surgery, abnormal stent migration occurred in nine patients (10.2%) within the first year. The migration sites included the hepaticojejunal anastomosis in four cases, the intrahepatic duct in three cases, and the remnant pancreatic duct in two cases.^9^ Since the migration site can cause pancreatitis, intestinal obstruction, liver abscess, or cholangitis, stent removal is recommended for symptomatic and asymptomatic cases where the stent has been retained for 18 months or more.

Stent retrieval methods include surgical, percutaneous, and endoscopic approaches. Among them, endoscopic retrieval is often attempted first due to its minimally invasive nature. However, there are no dedicated devices for the retrieval of migrated stents, and most endoscopists use several existing devices to remove them. Recently, the endoscopic introducer was developed to deliver endoscopic devices more safely and accurately. This device consists of a 7.2Fr outer sheath and a tapered inner sheath, which reduces the step between the guidewire and the sheath, allowing smooth insertion. The device is advanced over a guidewire to the target site; once positioned, the guidewire and inner sheath are removed, leaving the outer sheath in place. Devices up to 5.7Fr in diameter can then be inserted or removed through the outer sheath. A PubMed search (duration: 2015–2024) with the keywords “migrated stent,” “pancreas,” “pancreatic stent,” “sheath,” “device delivery system,” and “pusher tube” identified five cases of successful retrieval of migrated pancreatic stents using a device delivery system (Table 1). The median patient age was 78 years, including three men and two women. The stents were all made of plastic, and the device delivery system was inserted through the papilla in all cases. The endoscopic introducer was used in three cases, endoscopic retrograde cholangiopancreatography guided sheath (UMIDAS sheath cannula; Olympus) in one case, and a method utilizing an 8.5Fr bile duct stent pusher tube was employed in one case. Biopsy forceps were used in three cases and basket catheters in two cases. All cases were successfully managed without complications.

**TABLE 1:** List of previously reported cases of pancreatic duct stent removal through the papilla using a device delivery system.

Author	Age (y)	Sex	Etiology	Migrated sent Size/length/type	Symptoms	Device delivery system	Grasping device	Complications
Kato A.^4^	84	F	IPMN	7Fr/NA/plastic stent	Unknown	EndoSheather	Basket catheter	None
Higashimori A.^5^	55	M	CP	5Fr/NA/Plastic stent (fracture)	None	EndoSheather	Biopsy forceps	None
Matsumi A.^6^	80	M	Prevention for PEP	5Fr/3 cm/plastic stent	None	EndoSheather	Biopsy forceps	None
Ushio M.^7^	78	F	Prevention for PEP	NA/NA/plastic stent	Pancreatitis	UMIDAS sheath cannula	Biopsy forceps	None
Takahashi K.^8^	71	M	Prevention for PEP	5Fr/3 cm/plastic stent	None	8.5Fr stent pusher tube	Basket catheter	None

Abbreviations: CP, chronic pancreatitis; EndoSheather, manufactured by Piolax, Kanagawa, Japan; IPMN, intraductal papillary mucinous neoplasm; NA, not available; PEP, post‐endoscopic retrograde cholangiopancreatography pancreatitis; UMIDAS, sheath cannula manufactured by Olympus, Tokyo, Japan.

PubMed search (duration: 2015–2024) using the keywords “endoscopic ultrasound‐guided pancreaticogastrostomy” and “migrated stent” identified only one case of stent removal through ESCR. In that case, following PD, DBE was performed for pancreatic duct drainage but resulted in intestinal perforation, necessitating EUS‐PD. Subsequently, two plastic stents were regularly exchanged. However, one straight‐type plastic stent migrated into the pancreatic duct. Using a peroral pancreatoscope (POPS), the migrated stent was successfully retrieved via ESCR.^10^ In this case, due to the narrow pancreatic duct diameter, POPS insertion was challenging. Instead, the endoscopic introducer was used, and biopsy forceps were successfully used to remove the stent. This approach proved cost‐effective, and efficient and reduced procedural time (Table 2).

**TABLE 2:** Pancreatic stent removal via the endoscopy/ultrasonography‐guided route.

Author	Age	Sex	Etiology	Surgery	Symptoms	Migrated stent	Device	Grasping instrument	Complications
Takahashi S.^10^	68	M	Cholangiocarcinoma	PD	None	Straight type Trans‐jejunal plastic stent	SpyDS	Snare	None
Our case	64	F	Pancreatic carcinoma	PD	Abdominal pain	Straight type Internal plastic stent	EndoSheather	Biopsy forceps	None

Abbreviations: EndoSheather, an instrument manufactured by Piolax, Kanagawa, Japan; PD, pancreaticoduodenectomy; SpyDS, SpyScope DS 2 (Natick, Massachusetts, US).

This case report details the first report of the successful retrieval of an internal stent that migrated to a remnant pancreas after PD using an endoscopic introducer via ESCR. Traditionally, approaching the pancreatic duct in reconstructed intestines using DBE involves several steps, including advancing the scope to the pancreatojejunostomy, identifying the pancreatic duct anastomosis, and inserting the catheter. Each of these steps can be anatomically challenging and often preoperatively unpredictable. Utilizing ESCR is unaffected by these factors, allowing for a rapid approach to the pancreatic duct and a reduction in procedure time. Furthermore, in the present case, the method combined existing techniques and devices, making it universally applicable in enabling stent removal in a low‐cost, safe, and minimally invasive manner compared to open surgery. For cases where DBE‐ERCP is challenging or where procedure time needs to be minimized, this technique may be used as a viable treatment option. With further standardization and safety validation, ESCR has the potential to become a treatment option comparable to DBE.

## CONFLICT OF INTEREST STATEMENT

None.

## ETHICS STATEMENT

N/A
